# Interleukin-6 gene (*IL-6*): a possible role in brain morphology in the healthy adult brain

**DOI:** 10.1186/1742-2094-9-125

**Published:** 2012-06-13

**Authors:** Bernhard T Baune, Carsten Konrad, Dominik Grotegerd, Thomas Suslow, Eva Birosova, Patricia Ohrmann, Jochen Bauer, Volker Arolt, Walter Heindel, Katharina Domschke, Sonja Schöning, Astrid V Rauch, Christina Uhlmann, Harald Kugel, Udo Dannlowski

**Affiliations:** 1 Discipline of Psychiatry, School of Medicine, University of Adelaide, North Terrace, Adelaide, South Australia, 5005, Australia; 2 Department of Psychiatry, University of Marburg, Marburg, Germany; 3 Department of Psychiatry, University of Muenster, Muenster, Germany; 4 Department of Psychiatry, Psychosomatics and Psychotherapy, University of Wuerzburg, Wuerzburg, Germany; 5 Department of Psychosomatic Medicine and Psychotherapy, University of Leipzig, Leipzig, Germany; 6 Supramolecular and Synthetic Biology Group, School of Pharmacy and Molecular Sciences, James Cook University, Brisbane, QLD, Australia; 7 Department of Clinical Radiology, University of Muenster, Muenster, Germany

**Keywords:** Genetics, Inflammation, Interleukin 6, Neuroprotection, Voxel-based morphometry

## Abstract

**Background:**

Cytokines such as interleukin 6 (IL-6) have been implicated in dual functions in neuropsychiatric disorders. Little is known about the genetic predisposition to neurodegenerative and neuroproliferative properties of cytokine genes. In this study the potential dual role of several *IL-6* polymorphisms in brain morphology is investigated.

**Methodology:**

In a large sample of healthy individuals (*N* = 303), associations between genetic variants of *IL-6* (rs1800795; rs1800796, rs2069833, rs2069840) and brain volume (gray matter volume) were analyzed using voxel-based morphometry (VBM). Selection of single nucleotide polymorphisms (SNPs) followed a tagging SNP approach (e.g., Stampa algorigthm), yielding a capture 97.08% of the variation in the *IL-6* gene using four tagging SNPs.

Principal findings/results

In a whole-brain analysis, the polymorphism rs1800795 (−174 C/G) showed a strong main effect of genotype (43 CC vs. 150 CG vs. 100 GG; x = 24, y = −10, z = −15; F(2,286) = 8.54, p_uncorrected_ = 0.0002; p_AlphaSim-corrected_ = 0.002; cluster size k = 577) within the right hippocampus head. Homozygous carriers of the G-allele had significantly larger hippocampus gray matter volumes compared to heterozygous subjects. None of the other investigated SNPs showed a significant association with grey matter volume in whole-brain analyses.

**Conclusions/significance:**

These findings suggest a possible neuroprotective role of the G-allele of the SNP rs1800795 on hippocampal volumes. Studies on the role of this SNP in psychiatric populations and especially in those with an affected hippocampus (e.g., by maltreatment, stress) are warranted.

## Introduction

Inflammation is implicated in the etiology and pathophysiology of several brain pathologies (e.g., major depression [1-3], Alzheimer’s disease [4], and post-stroke depression [5]), as well as in cognitive aging [6] and mortality [7]. Specific markers of systemic inflammation such as cytokines have been identified as important mediators of neurodegenerative [8] and neuroplastic [9,10] processes relevant to neuropsychiatric disorders. Some of these proteins (e.g., interleukin 1 beta, interleukin 6, tumor necrosis factor) play a critical role in physiological CNS processes, such as cognitive function under immunologically unchallenged conditions [2,6].

Interleukin 6 (IL-6) is a cytokine that has demonstrated both neurodegenerative [4] and neuroprotective [11,12] properties. For example, increased levels of IL-6 are associated with neuropsychiatric conditions, such as depression [13] and Alzheimer’s disease [14]. In addition, first studies on the association between IL-6 and brain volume suggested a role of increased serum levels of IL-6 in brain atrophy during normal aging in conjunction with other cytokines [15]. In addition, an association between IL-6 levels and decreased hippocampal gray matter volume in middle-aged adults has recently been reported [16].

However, the hypothesis that IL-6 is mainly proinflammatory and neurodegenerative is challenged [17] with results supporting that this cytokine has several anti-inflammatory and immunosuppressive activities that may play a downregulating role in inflammatory conditions [17]. In addition, IL-6 may act as a developmental neurotrophic factor [18,19], and it has been shown to improve survival *in vitro* of several classes of neurons [20-22]. Moreover, it is suggested that IL-6 predominantly plays a protective role by improving survival of neurons in culture [21,23,24], protecting neurons from excitotoxic and ischemic insults [25-28], and promoting the growth of axons and consequently the number of synapses in a region [29-32]. Additionally, evidence shows that IL-6 may play a major role in promoting synaptic plasticity, LTP, and memory consolidation [33-35]. Furthermore, IL-6 is found to regulate survival of differentiated neurons and the development of astrocytes [36,37]. Overall, these findings from previous studies suggest that higher IL-6 levels may play a dual role with both neurodegenerative and neuroprotective biological functions.

The current evidence in humans relies on measures of IL-6 in serum and CSF, whereas limited research on the influence of genetic variants of IL-6 on brain pathology has been published. The *IL-6* gene is located on chromosome 7p21, and the GG genotype of the frequently studied *IL-6* promoter −174 C/G variation relates to higher levels of IL-6 compared to the CC genotype [38]. Although this single nucleotide polymorphism (SNP) has received a lot of attention in research in aging and longevity, the findings are inconclusively showing an association between the numbers of G alleles either with increased [39] or decreased [38] longevity depending on the study design, ethnicity, lifestyle, and cultural differences. Additional single nucleotide polymorphisms (SNPs), such as rs1800796, influence IL-6 expression (G allele carriers increase IL-6 plasma levels) [40] and are influenced by the presence of other polymorphisms (e.g., rs2069833, rs2069840) at this chromosomal locus [41]. However, these other genetic variants of *IL-6* have hardly been studied in brain function yet.

Further clarification of the biological role of genetic variants of *IL-6* in the human brain is needed to describe its multifunctional effects. In this study, we investigate the role of the *IL-6* gene in brain function and brain morphology by investigating the association between several genetic variants of interleukin 6 and brain morphology in healthy adult individuals. While this analysis is conducted in a whole-brain fashion, we expect genetic effects particularly in the hippocampus (HC) since this brain region has a critical role in normal brain function and several neuropsychiatric disorders. The HC region is a highly important structure for memory consolidation, and it has shown a strong susceptibility to stress and response to cytokines [42]. Specifically, several studies have shown that depression, post-traumatic stress disorder (PTSD), and childhood maltreatment are associated with smaller hippocampal volumes [42-44]. The role of genetic inflammatory biomarkers, such as IL-6, in these relationships is unclear.

This study aims at an improved understanding of the genetic background of the dual role of IL-6 in brain morphology and the hippocampal structure in particular. We hypothesize that *IL-6* polymorphisms are related to brain gray matter volumes, specifically in the hippocampus. The analysis will inform future studies in clinical psychiatric populations on the possible role and selection of genetic variants of *IL-6* for the study of hippocampal function in neuropsychiatric disorders.

## Material and methods

*Subjects.* Healthy subjects (*N* = 303) aged 18–65 of Central European ancestry participated in the study. Data were pooled from various studies conducted at the Department of Psychiatry, University of Münster, Germany, all employing the same MRI sequence on the same scanner. All included subjects were thoroughly investigated by experienced psychologists and were free from any lifetime history of psychiatric disorders according to DSM-IV criteria [45], as diagnosed with the SCID interview [46]. Exclusion criteria were scores ≥ 10 on the Beck Depression Inventory (BDI) [47], any neurological abnormalities, history of seizures, head trauma or unconsciousness, intake of any psychotropic medication, and the usual MRI contraindications. Six subjects had to be excluded because of anatomical abnormalities (abnormally enlarged ventricles) or strong movement artifacts discovered in the structural MRI images checked by visual inspection and identification as extreme outliers in the check data quality function of the VBM8 Toolbox. The remaining *N* = 297 scans (mean age 33.4 ± 11.7; *N* = 124 men, *N* = 173 women) were clear of such problems. Verbal intelligence was estimated by the Mehrfachwahl-Wortschatz-Intelligenztest (multiple-choice vocabulary intelligence test; MWT-B) [48]. See Table 1 for sample characteristics. The study was approved by the Ethics Committee of the University of Münster. After complete description of the study to the participants, written informed consent was obtained.

**` 1:** **Selection of single nucleotide polymorphisms within*IL-6*gene**

**Gene**	**Gene position**	**Total no. of SNPs (MAF ≥0.01)**	**No. of tagging SNP**	**Mean r**^**2**^	**Selected SNPs**	**Position**	**Function**	**Alleles**	**MAF HapMapCEU**	**Alleles captured**	**Prediction (STAMPA)**
*IL-6*	chr7: 22,731,750-22,738,790	43	22	0.991	rs1800795	22733170	5' near gene	CG	0.467 (G)	8	97.08%
					rs1800796	22732771	5' near gene	CG	0.043 (C)	3	
					rs2069833	22734189	intron	CT	0.475 (T)	8	
					rs2069840	22735097	intron	CG	0.317 (G)	1	

SNP, single nucleotide polymorphism; MAF, minor allele frequency; r^2^, linkage disequilibrium statistic [49]. MAF data relates CEU population from HapMap Phase II + III [50].

## Selection of polymorphisms and genotyping

The presently analyzed sequence of the *IL-6* gene comprising about 4.8 kb. We investigated genetic polymorphisms within this region as well as neighboring 5’- and 3’- segments containing possible gene regulatory elements including positions between 22,731,750 and 22,738,790 at chromosome 7p21. The investigated region contains 43 single nucleotide polymorphisms (SNPs) [50]. Applying a tagging SNP approach, we used various techniques to limit the number of SNPs assessed to the most relevant as follows. Initially, we constructed the linkage disequilibrium (LD) pattern of the CEPH population of the HapMap Phase II genotype data to identify tagging SNPs by an aggressive tagging approach (MAF > 1% and r^2^ > 0.8) using the Gevalt v2 software package [51]. Subsequently, we reduced SNP numbers by assessing the ability of limited numbers of the tagging SNPs to predict the total SNP population using the Stampa algorithm [52]. With this approach, 97.08% of the variation in the gene was captured using four tagging SNPs (rs1800795; rs1800796; rs2069833; rs2069840). The mean r^2^ of individual tagging SNPs in conjunction with one or more tagged SNPs was 0.991 (see Table 2 for details). While the SNPs rs1800795 and rs1800796 have been shown to directly regulate IL-6 expression, the other two SNPs (rs2069833, rs2069840) are non-coding variants [53]. The G allele of marker rs2069840 has shown to be associated with lower IL-6 plasma concentrations under a dominant model in a recently published cohort study [54].

**Table 2:** **Sample characteristics dependent on*IL-6*-174 C/G genotype**

	CC (*N* = 43)	CG (*N* = 150)	GG (*N* = 100)	p-value, according to χ²-test (df = 2) or ANOVA (F_2,290_)
Age	34.6 ± 12.3	34.0 ± 12.3	31.8 ± 10.4	0.25
Sex (m/f)	19/24	61/89	42/58	0.92
Verbal IQ^1^	119.1 ± 13.8	118.3 ± 12.1	118.1 ± 11.9	0.92
Education years	15.0 ± 2.4	14.8 ± 2.1	14.7 ± 2.1	0.82
STAI trait^2^	31.0 ± 6.7	32.8 ± 6.3	32.3 ± 6.8	0.32
BDI	2.2 ± 3.0	2.3 ± 2.7	2.4 ± 2.9	0.93

^1^Assessed with the Mehrfachwahl-Wortschatz test (multiple-choice vocabulary test; Lehrl, 1995), data from *N* = 230 subjects available. ^2^ State-Trait Anxiety Inventory, data from *N* = 258 subjects available. ^3^ Beck Depression Inventory, data from *N* = 283 subjects available.

Genotyping of four tagging *IL-6* SNPs was carried out following published protocols applying the multiplex genotyping assay iPLEX™ for use with the MassARRAY platform [55], yielding an overall genotyping completion rate of 98.9% [4/297 genotyping failures for rs1800795 and rs2069833 (99.0%), 5/297 for rs1800796 and rs2069840 (98.7%)]. Genotypes were determined by investigators blinded for the study.

Hardy-Weinberg equilibrium was fulfilled for all four SNPs, according to the program Finetti provided as an online source (http://ihg.gsf.de/cgi-bin/hw/hwa1.pl; Wienker TF and Strom TM) (exact test: rs1800795, *p* = 0.33; rs1800796, *p* = 1; rs2069833, *p* = 0.33; rs2069840, *p* = 0.80).

### MRI methods

*Voxel-based morphometry:* T1-weighted high-resolution anatomical images were acquired on a 3-Tesla scanner (Gyroscan Intera 3 T, Philips Medical Systems, Best, The Netherlands) with a 3D fast gradient echo sequence (Turbo Field Echo, TFE), TR = 7.4 ms, TE 3.4 ms, FA = 9°, two signal averages, inversion prepulse every 814.5 ms, acquired over a field of view of 256 (FH) × 204 (AP) × 160 (RL) mm, phase encoding in AP and RL direction, reconstructed to cubic voxels of 0.5 mm × 0.5 mm × 0.5 mm. The VBM8 toolbox (version 419; http://dbm.neuro.uni-jena.de/vbm) was used for preprocessing the structural images with default parameters. Images were bias-corrected, tissue classified, and normalized to MNI-space using linear (12-parameter affine) and non-linear transformations, within a unified model [56] including high-dimensional DARTEL normalization to the default DARTEL template provided with the VBM8 toolbox (resolution 1.5 × 1.5 × 1.5 mm). Gray and white matter segments were modulated only by the non-linear components in order to preserve actual GM and WM values locally (modulated GM and WM volumes), which results in a correction for total brain volume.

Homogeneity of gray matter images was checked using the covariance structure of each image with all other images, as implemented in the check data quality function. As described above, six extreme outliers showing anatomical abnormalities or movement artifacts were identified and excluded. The modulated gray matter images were smoothed with a Gaussian kernel of 8-mm FWHW. Group statistics were calculated with second level models using SPM8. For each SNP a separate full factorial model was conducted using genotype as the between-subjects factor. Age, education, and gender were added to the model as nuisance regressors. There was an upgrade of the scanner gradient system in 2008 *(“Master” Gradient System to “Quasar Dual” Gradient System).* Although the MRI sequence remained identical before and after the gradient system upgrade, we additionally modeled the scanner upgrade as regressors of no interest.

To control for multiple statistical testing within the entire brain, we maintained a cluster-level false-positive detection rate at *p* < 0.05 using a voxel-level threshold of *p* < 0.005 with a cluster extent (k) empirically determined by Monte Carlo simulations (*n* = 1,000 iterations). This was performed by means of the AlphaSim procedure, which accounted for spatial correlations between BOLD signal changes in neighboring voxels [57], implemented in the REST toolbox (http://restfmri.net/forum/index.php). The empirically determined cluster thresholds were k = 340 voxels. The anatomical labeling for the whole-brain data was performed by means of the widely used AAL Toolbox [58] and additionally by means of the Anatomy Toolbox [59]. The present sample had sufficient power (1-β = 80%) to detect relatively small effect sizes in a three-group ANOVA (f = 0.17) and in an allele-dose regression (*r* = 0.14), as calculated with G*Power [60].

## Results

**Figure 1:**
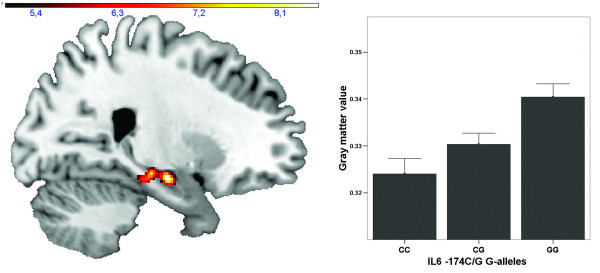
**Effect of*IL-6*rs1800795 (−174 C/G) genotype on brain morphometry.***Left panel*: Sagittal view through the right hippocampus (x = 24) depicting gray matter volume significantly modulated by the *IL-6* -174 C/G genotype, thresholded at *p* < 0.005, k = 340 (corresponding to *p* < 0.05, corrected on the cluster level for the entire brain), adjusted for age, education and gender. Color bar, F-value (df = 2,286); Right panel: Bar graph depicting gray matter concentration at x = 21, y = −12, z = −15 dependent on *IL-6* -174 C/G genotype

*rs1800795 (−174 C/G):* The whole-brain analysis yielded a strong main effect of genotype [43 CC vs. 150 CG vs. 100 GG), x = 24, y = −10, z = −15; F(2,286) = 8.54, p_uncorrected_ = 0.0002; p_AlphaSim-corrected_ = 0.002; cluster size k = 577, effect size f = 0.23 (Figure 1)]. According to the automated anatomical labeling, this cluster was located in the right hippocampus head, extending to the parahippocampal gyrus and the dorsal parts of the right amygdala. The Anatomy toolbox yielded similar localizations (peak effect was found in the cornu ammonis and subiculum area, extending to the laterobasal amygdala). There were no other areas in the entire brain surviving our corrected statistical threshold. Repeating this analysis with smoothing kernels of 6 mm or 10 mm still would yield significant findings.

According to post-hoc t-contrasts, subjects homozygous for the G-allele had significantly larger hippocampal gray matter volumes compared to heterozygous subjects, x = 24, y = −10, z = −15; t(286) = 3.88, p_uncorrected_ < 0.0001; p_AlphaSim-corrected_ < 0.001; cluster size k = 1210. Again, there were no other brain regions surviving the statistical threshold. However, there were no significant differences between heterozygous subjects and CC carriers in this model. Nonetheless, testing for allele-dose effects via regressing the number of rs1800795 (−174 C/G) G-alleles (0, 1, 2) on whole brain gray matter volume (again, including age, gender, education, and scanner gradient system as nuisance regressors) also yielded a significant cluster at a similar location in the right hippocampus, x = 21, y = −12, z = −15; t(287) = 3.82, p_uncorrected_ < 0.0001; p_AlphaSim-corrected_ = 0.001; cluster size k = 853, r = 0.22 (see Figure 1).

We further checked for interactions of the rs1800795 genotype and age as well as gender by modeling the interaction term in the three-group ANOVA model and the allele-dose regression. However, none of the interactions reached even a trend level of significance. Thus, the observed genotype effect on hippocampal gray matter volumes was comparable in men and women, and found across the entire age range.

*rs1800796, rs2069833, rs2069840*: No significant effects of these SNPs on hippocampus morphometry could be discerned in the whole-brain analysis.

## Discussion

This imaging genetics study investigated the association between the *IL-6* gene and brain morphology in a large cohort of healthy adult participants in a whole-brain analysis approach. Carriers of the G-allele of the *IL-6* genetic variant *rs1800795 (−174 C/G)* showed a significant association with larger hippocampal volumes on the right side in healthy subjects. This genotype effect was remarkably specific to the hippocampus, with no other structure surviving our statistical threshold corrected for the entire brain. The findings are suggestive of a neuroprotective role of the *IL-6* gene [rs1800795 (−174 C/G)] on hippocampal morphology. The IL6 genotype effect was found lateralized to the right. However, at a more lenient uncorrected statistical threshold, a similar genotype effect in the same direction could also be detected in the left hippocampus (*p* = 0.007, uncorrected, in the allele-dose model). Therefore, we discuss the observed effects for the hippocampus in general. The other investigated three SNPs showed no significant association with gray matter volume in our study. Since the SNPs 2069840 has been related to reduced IL-6 plasma levels, the lack of association in our study can be interpreted as consistent with the assumption that reduced plasma levels do not exert neuroplastic, neuroproliferative, or neuroprotective effects. In contrast, the marker rs1800796 showed no association with gray matter volume in our study, although, in a previous study, the G allele of this SNP has also been associated with higher IL-6 plasma levels [40]. Since these findings were derived from a clinical cohort of patients with diabetic nephropathy without data on brain morphometry, a direct comparison with our study is precluded.

Our study shows the strongest association between the *IL-6* genetic variant and HC volume, which has a number of critical functions under healthy and pathological conditions. It is part of a brain network including the dorsomedial and dorsolateral prefrontal cortex, the anterior cingulate cortex, and the amygdala dysregulated in major depression [61]. The HC is central to memory impairment, as seen in non-clinical samples [62] as well as in MDD [63]. Because the HC is a highly stress-sensitive brain region [64] and stress (psychological or psychosocial stress) is related to structural changes in the HC [65-67], atrophy of the HC has been described in imaging studies as a pathological neurobiological feature of depression associated with stress [68]. A meta-analysis of hippocampal volumes in patients with MDD confirmed that patients had hippocampal volumes approximately 4–6% smaller than matched control subjects in the left and right HC [69,70].

Although the possible role of IL-6 in brain morphology has not been extensively studied yet, our findings are in contrast with previous reports. These show associations between increased IL-6 plasma levels and reduced hippocampal volume in a relatively small study (*N* = 76) of middle-aged, relatively healthy individuals [16] in a study on first-episode psychosis [71], and in two studies investigating various brain areas and total brain volume, respectively, during aging [72,73]. Except in one study in relatively healthy individuals, these previous studies investigated individuals with underlying neuropsychiatric conditions. Variation in findings between these studies may be due to other methodological differences, such as the location of gray matter volume changes. While volume changes were located in the left HC in the study by Marsland et al. [16], and in various brain areas and total brain volume in the above-mentioned studies on aging [72,73], our results were specific to the HC. Another important difference between studies is the biological model of IL-6 effects in the brain. While those previous studies explain their findings using an inflammatory model in which it is proposed that IL-6 plays a proinflammatory role, the explanation of our study builds on the proven anti-inflammatory and immunosuppressive effects of IL-6 according to the well-established dual role of IL-6 [74]. A possible mechanistic explanation to support our finding that IL-6 was associated with increased HC volumes relates to the previously reported neuroproliferative effects of IL-6. For example, it has been shown that cytokines, including IL-6, despite being large molecules not freely passing through the blood–brain barrier, can enter the brain via various pathways (humoral, cellular, neural) [75] to exert their biological effects in the brain even under physiological conditions. More specifically, it has been shown that IL-6 primarily exerts its biological effects through a hexameric receptor ligand complex including the gp130 receptor [11] and the IL-6 receptor [76]. Distinct regions of gp130 activate specific signal-transduction pathways, such as the Janus kinase (JAK) signal transducer and activator of transcription (STAT), mitogen-activated protein kinase (MAPK)/cAMP responsive element-binding protein (CREB), Ras-MAPK, and PI-3 kinase (for review [77]). These pathways are related to neural plasticity by their ability to induce processes of neurogenesis, such as gliogenesis, neuronal differentiation, cAMP response element binding (cAMP), neural progenitor proliferation, and neuronal survival [77-79], and to enhance synaptic plasticity, LTP, and memory consolidation [33-35]. Through activation of these pathways, IL-6 has the ability to exert neuroprotective and neuroproliferative effects. In addition, IL-6 has been found to regulate survival of differentiated neurons and the development of astrocytes [36,37]. Some in-vitro studies show IL-6 release by activated microglia is a key inhibitor of neurogenesis by approximately 50%; others show IL-6 promoting differentiation of neural stem cells (NSCs) [10,80-82]. NSCs derived from rodent spinal cord show that IL-6 induces NSC proliferation via the JAK2/STAT3 and MAPK pathways [83]. Supporting a role of IL-6 in neuroproliferation is an *in-vivo* study showing that IL-6 knockout mice have reduced proliferating NSCs specifically in the HC, hence underlining the importance of IL-6 in cell proliferation and cell survival [84].

Despite mechanistic evidence and studies in humans for both pro- and anti-inflammatory effects of IL-6 in the brain, the role of IL-6 in the hippocampus remains to be clarified. Specifically, it is questionable that increased levels of IL-6 have purely degenerative effects since proliferative effects of IL-6 in the HC were demonstrated in an exercise study in mice: a wheel-running study in mice over 16 weeks showed that exercise increased IL-6 levels in the HC, whereas other cytokines such as TNF and IL-1ra decreased during exercise [85]. These results suggest that an upregulation of IL-6 could have anti-inflammatory effects and be neuroprotective in the cytokine milieu of the HC, and thereby IL-6 may buffer cognitive decline through exercise-induced changes in the HC milieu.

Translating these findings into a human study, one could argue that peripherally increased IL-6 levels could be interpreted as an anti-inflammatory activity rather than a proinflammatory state. Hence, previously observed correlations between increased plasma levels of IL-6 and decreased HC volumes could alternatively be interpreted as an anti-inflammatory response of IL-6 to other increased cytokines such as TNF and IL-1beta. Indeed, both cytokines have previously been shown to be associated with hippocampal volumes (TNF) [86] and with increased white matter hyperintensities (IL-1beta) [87] in healthy individuals. In such a case, IL-6 would only be a marker of a global inflammatory process, and reduced brain volume might primarily be induced by proinflammatory cytokines such as TNF and IL1-beta.

In light of these studies suggesting effects of IL-6 on various mechanisms subserving neuroproliferation and assuming that the carriers of the G-allele of the *IL-6* polymorphism rs1800795 (−174 C/G) in our sample have increased IL-6 levels as previously reported, it can be suggested that in our imaging study, this particular SNP might exert neuroprotective effects on the HC via increased IL-6 levels, hence the observed increased gray matter volume.

Our study has strengths and limitations. We were able to employ the genetic analysis in a large imaging sample using a cohort of carefully selected and well-characterized healthy individuals. For future studies, clinical measures such as hypertension or BMI could be useful covariates when investigating genetic inflammatory biomarkers such as IL-6; however, the relevance of hypertension might be of greater relevance in clinical samples than in our healthy cohort. Our discussion is based on the assumption that larger gray matter values in the hippocampus correspond to better function. Albeit reduced hippocampal volumes are consistently found in neuropsychiatric disorders, the relation of volume and function remains to be established more firmly. Although no protein data were available to validate the well-described upregulation of IL-6 by the SNP rs1800795 (−174 C/G), our study is the first genetic study investigating the association between the *IL-6* gene and brain morphometry, and the HC in particular. Future genetic imaging studies would benefit from additional protein data. Moreover, a clinical control group with a psychiatric disorder such as depression or psychosis/schizophrenia might add knowledge on the dual role of the *IL-6* gene in health and disease states. Another important consideration for interpreting these results is related to the lack of a cutoff of level of IL-6 defining normal, increased, and decreased peripheral IL-6 levels, limiting the interpretation of physiological and pathological brain conditions. The LD indices indicate complete LD (D’ = 1) for the correlation of all four marker combinations (except D’ = 0.993 for rs1800795 x rs2069833), which indicates that the reported findings are not explained by relevant SNP correlations.

## Conclusion

This imaging genetic study suggests the *IL-6* genetic variant rs1800795 (−174 C/G) as a biomarker of hippocampal morphometry. This genetic variant may exert neuroprotective effects on hippocampal volume in healthy individuals. Replication in independent and clinical samples is warranted.

## Competing of interests

KD has received speaker fees from Pfizer, Lilly, and Bristol-Myers Squibb; she has been a consultant for Johnson & Johnson and has received funding by Astra Zeneca. All other authors declare no conflicts of interest.

## Authors’ contributions

BTB conceived and proposed the genetic analysis, and wrote the first draft together with UD. UD performed the imaging and imaging-genetics analysis, and drafted parts of the manuscript. CK contributed to the design of the study, oversaw recruitment of participants, and contributed to the draft manuscript. DG contributed to the recruitment and assessment of participants. TS contributed to the study design and the interpretation of the results. EB contributed to the genetic analyses of the results. PO, SS, AV, and CU carried out the recruitment, assessment, and measurements of participants. WH, JB, and HK oversaw and conducted the imaging MRI component of the study. KD contributed to data of participants, and oversaw DNA collection and contributed intellectually to a draft of the manuscript. VA contributed intellectually to the content of the manuscript. UD oversaw, conceived the imaging study, and contributed to the writing of the MS. All authors contributed intellectually to the manuscripts and approved the final versions of the manuscript.
